# Effects of diversified volatile profiles on olfactory orientation of flea beetles *Phyllotreta* spp. and the diamondback moth *Plutella xylostella*

**DOI:** 10.1111/plb.13722

**Published:** 2024-09-24

**Authors:** J. K. Mäkinen, S. Saussure, H. Ruhanen, E. Räty, J. D. Blande

**Affiliations:** ^1^ Department of Environmental and Biological Sciences University of Eastern Finland Kuopio Finland; ^2^ Natural Resources Institute Finland (Luke) Jokioinen Finland

**Keywords:** Diamondback moth, flea beetle, olfactory orientation, strip cropping, volatile organic compounds

## Abstract

- This study investigated the effect of mixing volatile organic compounds (VOC) emitted by host and non‐host plants on the orientation of key pests of Brassicaceae. The study aimed to understand how these mixed VOCs influence pest behaviour, which could help in tailoring pest management strategies.
- The orientations of flea beetles, *Phyllotreta* spp., and the diamondback moth (DBM), *Plutella xylostella*, towards cabbage VOCs mixed with faba bean VOCs were assessed using Y‐tube olfactometry. The pests' preferences were measured to determine if the presence of faba bean alongside cabbage altered their olfactory orientation compared to cabbage alone.
- Flea beetles showed a preference for cabbage VOCs alone over the cabbage–faba bean VOC mix. For DBM, no significant preference was observed between cabbage alone and the cabbage–faba bean mix. Previous findings indicated that faba bean attracts DBM, and in this study the mixture of cabbage and faba bean appeared to be more attractive than cabbage alone.
- The results indicate that faba bean VOCs can deter flea beetles from cabbage, potentially offering a pest management strategy. However, the effect on DBM was inconclusive, with no clear preference observed. This suggests that while faba bean VOCs may influence pest orientation, their effectiveness varies among different pest species. Additionally, herbivore damage to cabbage leaves did not appear to influence the odour‐guided orientation of either pest, irrespective of the presence or absence of faba bean.

## Graphical abstract

Diversifying the crop volatile organic compound profile can alter pest orientation: flea beetles were less attracted to chemical cues of the cabbage–faba bean mix than to those from cabbage alone, while diamondback moths remained equally attracted to both cues.

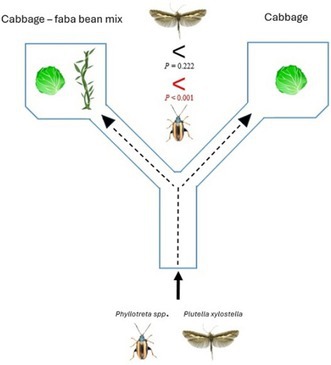

## INTRODUCTION

Herbivorous insects are major pests in agricultural settings, and are estimated to cause an 18% loss in crop yield globally (Wyckhuys *et al*. 2023). Pesticide use has been the prevalent method of pest control since the development of synthetic pesticides in the early 20th century (Tudi *et al*. 2021). Despite representing important tools in agricultural pest control and provision of food security, these chemicals have detrimental effects on non‐target organisms, such as natural enemies, surrounding ecosystems, and also on human health (Geiger *et al*. 2010; Boedeker *et al*. 2020). Due to these unintended negative effects, more sustainable solutions for pest control are being developed. These solutions can be deployed alone or in combination, such as choosing pest insect resistant plant varieties, or installing physical barriers with nets or covers over the crops.

One particular pest control method relies on using plant‐emitted volatile organic compounds (VOCs) to manipulate orientation of the pests. Pest insects utilize vision and olfaction as the main sensory modalities to guide orientation towards target plants for nutrition or oviposition (Brévault & Quilici 2010). Olfaction enables long‐distance foraging for suitable host or food plants, because VOCs are carried by wind. Plant‐emitted VOCs are diverse, with more than 1700 such compounds known to be emitted by both angiosperms and gymnosperms (Picazo‐Aragonés *et al*. 2020). Even diverse blends provide valuable cues for insects, but it is not completely understood how insects filter out relevant information from such complex blends. In some cases, the presence of a target plant is indicated by just a few VOCs (Conchou *et al*. 2019), whereas in other cases distinct ratios of compounds in a blend provide important information (Bruce & Pickett 2011). Furthermore, VOC emissions are usually altered after pest damage, which can signal to herbivores that the plant is already under attack. These changes to plant VOC emissions, known as herbivore‐induced plant volatiles (HIPVs), have been widely documented to attract natural enemies and thus mediate indirect defence (Himanen *et al*. 2015). However, HIPVs may also increase the apparency of a plant and act as attractive cues to pest insects (Ameye *et al*. 2018) or, alternatively, they may directly repel pests that could perceive HIPVs as an indicator of competition for their offspring (Reyes‐Prado *et al*. 2020). Therefore, manipulating VOC cues by: (i) masking specific VOCs used by pests to orient towards their host plants, i.e., mixing the informative volatiles within a larger blend to create a new type of odour and confuse the pests, or (ii) by selecting crops that emit pest‐repelling VOCs, are potential methods for controlling pest numbers and limiting crop damage (Hooks & Johnson 2003). Diversified farming practices, such as intercropping, companion cropping, or push‐pull integrated pest management, aim at using plant traits, including volatiles, to sustainably protect crops. For example, strip cropping, a form of intercropping, is an agricultural technique where two or more crop types are planted or sown in strips in the same field area. Such mixed crop types diversify the emitted VOCs, potentially making it harder for pest insects to orientate towards hosts (Huss *et al*. 2022).

Flea beetles (*Phyllotreta* spp.; Coleoptera: Chrysomelidae) are herbivorous insects and a major pest of cruciferous plants. They are named for their ability to jump like fleas when disturbed and are known for their distinctive appearance, which includes a shiny, metallic body often with yellow stripes. Flea beetles are found in many different habitats worldwide, but are particularly common in agricultural and horticultural settings, where they can cause significant damage to brassicaceous vegetable and oilseed crops in the mustard family (Brassicaceae) (Weber *et al*. 2022). Flea beetles feed on plant leaves and stems both during their adult and larval stages. The most serious damage is caused at the seedling stage, about 2 weeks after the seedling has emerged (Knodel 2017). Feeding can then reduce photosynthesis and yield, cause necrosis, and ultimately death of the plant (Tengfei *et al*. 2022).

The diamondback moth (*Plutella xylostella*; Lepidoptera: Plutellidae) is also a major pest insect of brassicaceous vegetables worldwide, with annual management costs estimated in the billions of dollars (Philips *et al*. 2014). Larvae feed on the leaves of plants, reducing photosynthesis and potentially leading to plant death. They are particularly problematic in areas where insecticide resistance is common (Philips *et al*. 2014). Diamondback moths cannot currently overwinter at northern latitudes and are carried there by wind, which increases the annual variations in their abundance (Dancau *et al*. 2018). However, as global temperatures continue to rise, moth overwintering in Nordic countries may become increasingly common, potentially worsening crop damage levels.

Flea beetles and diamondback moths can both be managed through a variety of pest control methods, including host plant resistance, biological control, cultural controls, behavioural management, and by the use of insecticides (Philips *et al*. 2014). However, it has been shown that none of the control methods alone are adequate, and insecticide misuse, which has caused resistance in both of these pests, will likely continue to weaken their efficacy (Philips *et al*. 2014; Willis *et al*. 2020; Shen *et al*. 2023). Therefore, it is important to develop a crop protection approach that utilizes multiple complementary techniques to deliver a more sustainable outcome.

This study aimed to assess: (i) the effect of mixing odour sources from a host plant, cabbage, and a non‐host plant, faba bean, on the orientation of flea beetles and DBM, and (ii) the effect of pre‐existing foliar damage to the host plant on the pest orientation. For *Brassica* pest control based on chemical ecology, it is important to understand how both flea beetles and DBM orientate towards VOC blends of host and non‐host plants presented alone or in combination, and if pre‐existing damage to the host plant modifies the pest orientation. Flea beetles and DBM were chosen because of their agronomic importance and the high densities observed in a previous field experiment within a cabbage–faba bean cropping system (Mäkinen *et al*. 2023). This complementary laboratory study investigated whether the combinations of plant odours present in this crop combination affect the pest orientation, and if these have potential for future use in pest management. We hypothesized that: (i) mixing VOCs from the host plant with those of a non‐host plant would reduce the efficiency of VOC‐mediated orientation of flea beetles and DBM towards cabbage, and (ii) pre‐existing foliar damage would influence the orientation of these two pests, potentially disrupting the masking effect of faba bean.

## MATERIAL AND METHODS

### Plants and insects

#### Cabbage and faba bean

Cabbage (*Brassica oleracea* var. *capitata* ‘Lennox F1’) and faba bean (*Vicia faba* ‘Aunus’) plants were sown in separate plastic pots, with one plant per pot. The soil was a mixture of peat, soil, and sand (ratio 3:1:1), and the plants were watered daily and fertilized twice a week with 0.2% Kekkilä Taimi‐superex nutrient solution (N‐P‐K: 19‐5‐20). All the plants used in experiments were 4–6 weeks old. In order to damage cabbages, plants were infested with 10 diamondback moth (DMB) larvae for 24 h, and the larvae were removed before initiating experiments.

#### Flea beetles (*Phyllotreta* spp.) (Coleoptera: Chrysomelidae)

During spring and throughout the summer, adult flea beetles were caught from a field site in Kuopio, Finland (62°55′44.9″ N, 27°37′27.5″ E) and transported to the laboratory where they were kept in transparent plastic cages for 24 h before experiments. During the 24 h waiting period they were provided with water absorbed into cotton wool and with cabbage plants (*Brassica oleracea* var. *capitata* ‘Lennox F1’) as a food source. Flea beetles were kept in an insect‐rearing room, with a day/night cycle of 16:8 h and a temperature between 20 and 25 °C. The flea beetles belong to the genus *Phyllotreta*, commonly known as cabbage beetles (Brust *et al*. 2017), six species of which are present in Finland and share similar ecological niches (Knodel 2017). The six species are *Phyllotreta striolata*, *P. undulata*, *P. nemorum*, *P. flexuosa*, *P. armoraciae*, and *P. vittula*. In this study, individuals were obtained directly from the field and were not identified to species level. Since a single *Phyllotreta* sp. is rarely responsible for crop damage in the field, the complex of naturally occurring flea beetles was used and enabled assessment of the general orientation of flea beetles towards the VOCs of a cabbage–faba bean cropping system. From here on, “flea beetles” indicates a natural population and does not include accurate identification to species level. Males and females were also not separated because both sexes consuming leaves and females oviposit into the soil.

#### Adult diamondback moth (*Plutella xylostella*) (Lepidoptera: Plutellidae)

Adult DBM were reared at the Kuopio campus of the University of Eastern Finland. The population originated from the Netherlands, but new individuals from populations collected in Finland have been added each year to diversify the gene pool. DBM were kept in the same room as the flea beetles and under the same conditions. Only females were used in the experiment, as they actively seek out host plant species to locate mating partners and egg‐laying sites (Couty *et al*. 2006).

### Experimental setup

#### Odour sources

The odour sources used in both the flea beetle and DBM experiments (Table 1) were as follows: undamaged cabbage (UC), undamaged faba bean (F), undamaged cabbage–undamaged faba bean mix (UCF), damaged cabbage (DC) and damaged cabbage–undamaged faba bean mix (DCF). The odour combinations bioassayed for the flea beetles and DBM differed slightly. This was mostly because flea beetles were collected directly from the field and because their availability limited the number of tests that could be conducted with robust replication.

**Table 1:** Replicate number and the corresponding plant pairs in olfactory orientation of flea beetles in Y‐tube olfactometer.

replicate number	treatments in flea beetle orientation experiment	treatments in DBM orientation experiment
1	Faba bean F and Undamaged cabbage UC	Faba bean F and Undamaged cabbage UC
2	Undamaged cabbage–faba bean mix UCF and Undamaged cabbage UC	Damaged cabbage DC and Undamaged Cabbage UC
3	Undamaged cabbage–faba bean mix UCF and Damaged cabbage DC	Undamaged cabbage–faba bean mix UCF and Undamaged Cabbage UC
4	Damaged cabbage–faba bean mix DCF and Undamaged cabbage UC	Undamaged cabbage–faba bean mix UCF and Damaged cabbage DC
5		Damaged cabbage–faba bean mix DCF and Damaged cabbage DC

#### Orientation experiment

The orientation of insects to combinations of the various odour sources described above was tested using Y‐tube olfactometry. The Y‐tube was situated within a green open‐top box equipped with holes on both sides, facilitating insertion of the tubes that carried airflow from two glass desiccators containing the potted plants under examination. Each glass desiccator was equipped with a perforated cap on its lid, connected to a tube for introducing clean air into the desiccator. A similar perforated cap on the side of the desiccator allowed the tube carrying the air exiting the desiccator to connect to the Y‐tube. Teflon and masking tape were used to seal the desiccators, with Teflon tape underneath the masking tape to prevent VOCs from the masking tape entering the desiccators. A lamp placed above the box served as a light source, with other light sources excluded throughout the experiment. Clean air was sourced from an AADCO 474‐30 Ultra High Purity Zero Air Generator (ZAG). The airflow rates in the tubes connected to the branches of the Y‐tube ranged from 340 to 360 ml·min^−1^. The difference in airflow between the tubes leaving the desiccators was less than 10 ml·min^−1^ for each treatment. Airflow calibration was conducted using a mini‐BUCK bubble calibrator (M‐5; A.P. Buck, Orlando, FL, USA). For both flea beetles and DBM, a system control was implemented using empty desiccators to ensure that the equipment itself did not influence insect orientation. Plants were changed after every 10 insects assayed, at which point the location of the odour sources was switched to avoid directional bias in the Y‐tube. Each olfactory choice test was repeated with 50 different insects. The system control tests with empty desiccators were performed with 20 different insects. Before commencing experiments, the desiccators were cleaned with 70% ethanol and left to dry. The Y‐tubes were also cleaned and heated in an oven at 120 °C for 1 h before cooling to room temperature.

The insects were released into the Y‐tube from a small plastic tube with a mesh‐covered end. Each insect was given 5 min to orientate within the Y‐tube. The odour sources were the only available cues for the insects to utilize for orientation, no visual cues were provided. A choice was recorded if an insect moved more than two‐thirds of the way along an arm of the Y‐tube. If insects did not pass the designated point, it was recorded as a “no choice”. After each insect, the Y‐tube was rotated 180°, i.e., left arm of the tube would be the right arm for the next insect. This was done to exclude the possibility of the insects following each other's odour trails. After ten insects, the Y‐tube was replaced with a clean one. Each insect was used once.

#### VOC collection in flea beetle experiment

Collection of VOCs was done for each odour combination for the experiment with flea beetles. After the assigned 10 insects had been used in the Y‐tube experiment, VOCs were collected from both desiccators. This was done to gain precise information on the odour sources to which each group of insects was exposed and enable interpretation of deviations in behaviour for the different odour source pairings. VOCs were collected five times for each treatment, giving a total of 40 samples (Table 1). We did not collect VOC emissions in the DBM experiment. Sample tubes (Markes International, Llantrisant, UK) filled with Tenax TA 60/80 adsorbent were used to collect the samples. The sample tube was connected to the desiccator with a plastic coupler and the other end of the Tenax tube was connected to a vacuum pump that pulled air through the tubes at 240 ml·min^−1^. Air was pushed into the desiccators at 340–360 ml·min^−1^, and air flows were calibrated with a mini‐BUCK bubble calibrator. Samples were collected for 30 min. After collection, the ends of the sample tubes were closed with brass caps, wrapped in aluminium foil, and transferred to a refrigerator to await analysis with a gas chromatography‐mass spectrometer.

### Gas chromatography‐mass spectrometry (GC‐MS)

Samples and standards were run on a GC‐MS equipped with a thermodesorption unit (GCMS‐QP2020 and TD‐30R; Shimadzu, Kyoto, Japan). Compounds were desorbed at 300 °C for 10 min, cryofocused at −20 °C, and injected into a gas chromatin ZB‐5MS plus capillary column (60 m × 0.25 mm, membrane thickness 0.25 μm). The carrier gas was helium. The column temperature was first held at 40 °C for 1 min; thereafter the temperature was programmed to increase from 40 to 125 °C at 5 °C·min^−1^ and finally to 250 °C at 10 °C·min^−1^.

The chromatograms were analysed with the ChemStation Data Analysis software. The compounds contained in the samples were identified using external standards, one series for terpenoids and one for green leaf volatiles, which were run twice to determine consistency. The standards contained a total of 19 monoterpenes, 9 sesquiterpenes, and 9 green leaf volatiles (Sigma‐Aldrich, Munich, Germany). The rest of the compounds were identified tentatively using the Wiley 275 library.

### Statistical analysis

Data from the orientation experiments of both flea beetles and DBM were analysed with one sample binomial tests. VOC data from the flea beetle experiment were analysed in three different ways. First, we analysed the total VOCs emitted in nanograms for each of the treatments represented in Table 1. This analysis was done with one‐way anova, with Tukey's HSD as a posth‐oc test, and the data were transformed with a Reciprocal Transformation to achieve normality. Second, differences in the individual compounds emitted by each plant combination were analysed (Table 2) using the non‐parametric independent samples Kruskal‐Wallis test. We focused on the specific individual compounds emitted by the plants, which is why we did not analyse categories of volatiles, such as GLVs, across the treatments. The significance values were adjusted with the Bonferroni correction for multiple tests. Third, the VOC data were also analysed pairwise, i.e., comparison of the odour sources in each choice scenario used during the flea beetle assays. This pairwise analysis was done using the non‐parametric Mann–Whitney U‐test. Due to an unexpected result in flea beetle orientation in the undamaged cabbage–faba bean combination, a correlation coefficient was calculated to determine if the total VOC emissions of undamaged cabbage correlated with the flea beetle orientation towards undamaged cabbage.

**Table 2:** VOC emissions (ng) from flea beetle experimental treatments and significant differences in pairwise analysis.

	UC (n = 15)	F (n = 5)	UCF (n = 10)	DC (n = 5)	DCF (n = 5)	test statistic	*P*‐value
Non‐oxygenated monoterpenes							
α‐Thujene	5.07 ± 0.96 ab	1.61 ± 1.19 ab	6.39 ± 0.90 a	3.78 ± 0.46 ab	0.63 ± 0.12 b	13.197	**0.010**
α‐Pinene	2.52 ± 0.45 ab	0.94 ± 0.40 ab	3.59 ± 0.69 a	2.53 ± 0.57 ab	0.99 ± 0.17 b	12.988	**0.011**
Camphene	0.02 ± 0.01	0.00 ± 0	0.03 ± 0.03	0.00 ± 0	0.00 ± 0	2.316	0.678
Sabinene	11.98 ± 2.20 ab	4.99 ± 3.54 b	25.75 ± 2.70 a	13.49 ± 2.10 ab	3.37 ± 1.06 ab	16.788	**0.002**
β‐Pinene	1.91 ± 0.72	0.52 ± 0.33	1.43 ± 0.26	1.36 ± 0.16	0.45 ± 0.08	8.556	0.073
β‐myrcene	5.30 ± 0.94 ab	1.78 ± 1.31 ab	5.22 ± 0.83 a	4.27 ± 0.67 ab	0.99 ± 0.32 b	12.962	**0.011**
3‐Carene	0.15 ± 0.04	0.16 ± 0.06	0.05 ± 0.26	1.24 ± 0.53	0.16 ± 0.09	7.674	0.104
α‐Terpinene	0.18 ± 0.06	0.06 ± 0.06	0.22 ± 0.10	0.10 ± 0.04	0.03 ± 0.02	4.871	0.301
Limonene	8.84 ± 1.47 ab	3.10 ± 2.39 ab	12.46 ± 1.48 a	7.87 ± 1.33 ab	2.98 ± 0.58 b	12.643	**0.013**
(*E*)‐β‐Ocimene	0.05 ± 0.04	0.33 ± 0.23	1.07 ± 0.50	0.00 ± 0	2.74 ± 2.67	10.345	0.035*
γ‐Terpinene	0.41 ± 0.13	0.16 ± 0.16	0.61 ± 0.14	0.27 ± 0.07	0.05 ± 0.03	9.397	0.052
Oxygenated monoterpenes							
1,8‐Cineole	8.35 ± 1.27 ab	3.72 ± 2.02 ab	11.46 ± 3.07 a	5.84 ± 1.20 ab	2.74 ± 0.24 b	13.051	**0.011**
(*Z*)‐sabinene hydrate	1.38 ± 0.22 a	0.45 ± 0.45 ab	0.93 ± 0.19 ab	1.35 ± 0.37 ab	0.11 ± 0.06 b	12.678	**0.013**
Linalool	0.00 ± 0	0.00 ± 0	0.34 ± 0.24	0.00 ± 0	0.82 ± 0.82	5.271	0.261
Menthol	0.48 ± 0.19	0.06 ± 0.06	0.75 ± 0.35	0.24 ± 0.06	0.10 ± 0.06	4.334	0.363
Terpinen‐4‐ol	0.31 ± 0.05 ab	0.11 ± 0.10 ab	0.31 ± 0.05 ab	0.46 ± 0.05 a	0.12 ± 0.08 b	11.096	**0.026**
Homoterpenes							
(*E*)‐DMNT	0.10 ± 0.05	0.00 ± 0	0.41 ± 0.30	0.23 ± 0.14	0.00 ± 0	2.509	0.643
Sesquiterpenes							
(*E*)‐Caryophyllene	0.11 ± 0.07 b	1.53 ± 0.68 abc	4.18 ± 0.44 a	0.00 ± 0 c	1.67 ± 0.49 abc	30.373	**<0.001**
α‐Humulene	0.00 ± 0 b	0.00 ± 0 b	0.91 ± 0.40 a	0.00 ± 0 b	0.30 ± 0.30 b	10.796	**0.029**
Green leaf volatiles							
(*Z*)‐3‐hexen‐1‐ol	0.29 ± 0.20 b	4.20 ± 3.45 ab	5.18 ± 2.98 ab	1.08 ± 0.84 ab	9.85 ± 6.47 a	10.457	**0.033**
1‐hexanol	0.19 ± 0.09	0.22 ± 0.11	1.20 ± 0.47	0.15 ± 0.08	0.91 ± 0.48	9.398	0.052
(*E*)‐2,4‐hexadienal	0.08 ± 0.08	0.00 ± 0	0.00 ± 0	0.00 ± 0	0.01 ± 0.01	3.285	0.511
1‐Octen‐3‐ol	0.23 ± 0.10	0.14 ± 0.04	0.75 ± 0.31	0.16 ± 0.12	0.59 ± 0.25	9.983	0.041*
2‐Methyl‐2‐hepten‐6‐one	7.67 ± 1.87	3.99 ± 0.60	12.46 ± 4.03	6.77 ± 0.81	3.86 ± 0.82	6.441	0.169
(*Z*)‐3‐hexenyl acetate	0.90 ± 0.90 b	0.00 ± 0 c	21.95 ± 13.9 abcd	0.00 ± 0 c	21.90 ± 10.59 a	16.866	**0.002**
1‐Hexyl acetate	0.00 ± 0	0.00 ± 0	0.20 ± 0.14	0.00 ± 0	0.62 ± 0.62	5.271	0.261
2‐Ethyl hexanol	7.31 ± 1.38 ab	5.92 ± 1.13 ab	12.03 ± 2.53 a	11.17 ± 4.39 ab	3.57 ± 0.46 b	12.157	**0.016**
Nonanal	64.79 ± 23.07	30.91 ± 4.78	132.51 ± 67.92	30.84 ± 6.98	24.41 ± 4.67	0.835	0.934
Isothiocyanates							
Allyl isothiocyanate	0.28 ± 0.27	0.00 ± 0	0.05 ± 0.05	0.58 ± 0.31	1.22 ± 0.55	11.274	0.024*
Other nitrogen‐containing compounds							
Estragole	0.00 ± 0	0.00 ± 0	0.04 ± 0.04	0.00 ± 0	0.00 ± 0	3.000	0.558
Other compounds							
Methylsalicylate	0.14 ± 0.08	0.07 ± 0.03	0.46 ± 0.22	0.03 ± 0.03	0.03 ± 0.03	7.013	0.135
Geranyl acetone	3.06 ± 0.49	2.94 ± 0.21	4.34 ± 1.18	3.03 ± 0.35	2.15 ± 0.36	2.963	0.564

Pairs were UC–F, UCF–UC, UCF–DC and DCF–UC. Bold values in the *P*‐value column indicate statistical significance (*P* < 0.05). An asterisk (*) denotes that the value is no longer significant after applying the Bonferroni correction for multiple comparisons.

When two values of the same compound share the same alphabetical notation, the difference between them is not considered significant. However, when the alphabetical notations differ, the values are deemed significantly different. For example, in a‐thujene, the UC value of 5.07 and the *F* value of 1.61 both have the letters 'ab' following them, indicating no significant difference. In contrast, the UC value of 5.07 and the DCF value of 0.63 have different notations, suggesting a significant difference.

## RESULTS

### Olfactory orientation of flea beetles

**Fig. 1:**
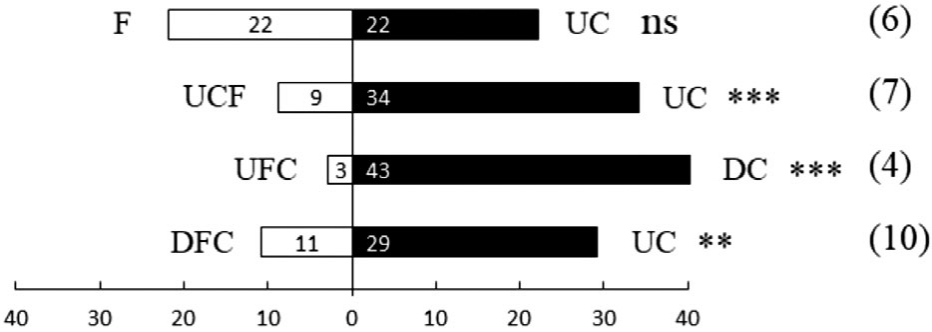
Olfactory orientation of flea beetles in Y‐tube olfactometer. Fifty insects were tested per combination, the number on the bar indicates number of insects that selected that odour source. The number in parentheses is number of individuals not making a choice. Asterisks indicate significance: ns = *P* > 0.05, *P ≤ 0.05, **P ≤ 0.01, ***P ≤ 0.001.

When given a choice of UC or F, there appeared to be no significant difference in the orientation of flea beetles (N = 44, standardized *Z* < 0.001, SE = 3.317, *P* = 1.000; Fig. 1). High degree of correlation was observed in released total amount of VOCs in UC and number of choices towards this option in the UCF crop combination (Fig. 4). However, significant differences in orientation emerged in all other scent combinations. Indeed, when offered a choice between UC and the combined VOC blend of UCF, flea beetles oriented towards the UC with a significant difference (N = 43, standardized *Z* = 3.660, SE = 3.279, *P* < 0.001; Fig. 1). When presented with the option of UCF versus DC, flea beetles oriented towards DC with a significant difference (N = 46, standardized *Z* = 5.750, SE = 3.391, *P* < 0.001; Fig. 1). Finally, when given the choice between DCF and UC, flea beetles oriented significantly more towards UC (N = 41, standardized *Z* = 2.811, SE = 3.202, *P* = 0.005; Fig. 1).

### Olfactory orientation of DBM

**Fig. 2:**
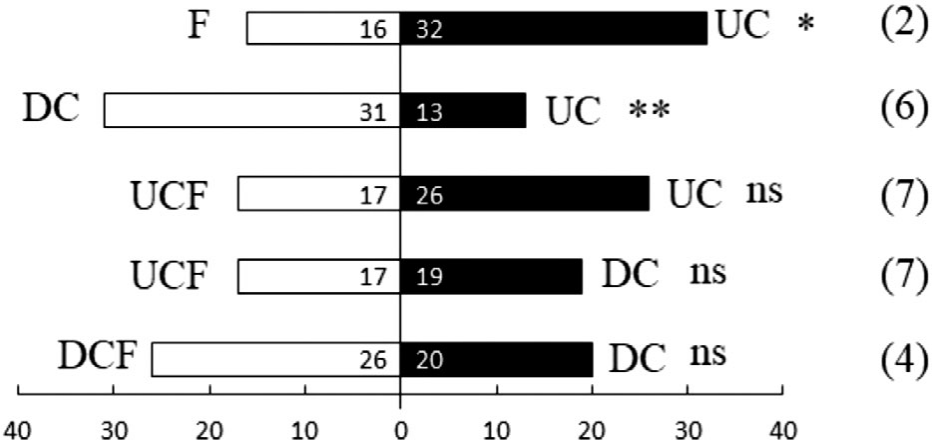
Olfactory orientation of DBM in Y‐tube olfactometer. Fifty insects were tested per combination, the number on the bar indicates number of insects that selected that odour source. The number in parentheses is number of individuals not making a choice. Asterisks indicate significance: ns = *P* > 0.05, *P ≤ 0.05, **P ≤ 0.01, ***P ≤ 0.001.

Given a choice of UC or F, DBM oriented significantly more often towards UC (N = 48, standardized *Z* = 2.165, SE = 3.464, *P* = 0.03; Fig. 2). When presented with DC and UC, DBM oriented significantly more often towards DC (N = 44, standardized *Z* = 2.563, SE = 3.317, *P* = 0.01; Fig. 2). However, no significant preferences were observed in the orientation of DBM when presented with choices between UCF and UC (N = 43, standardized *Z* = −1,220, *P* = 0.222; Fig. 2), DC and UCF (N = 36, standardized *Z* = −0.167, SE = 3.000, *P* = 0.868; Fig. 2), or DC and DCF (N = 46, standardized *Z* = −0.737, SE = 3.391, *P* = 0.461; Fig. 2).

### VOCs collected in the flea beetle orientation experiment

**Fig. 3:**
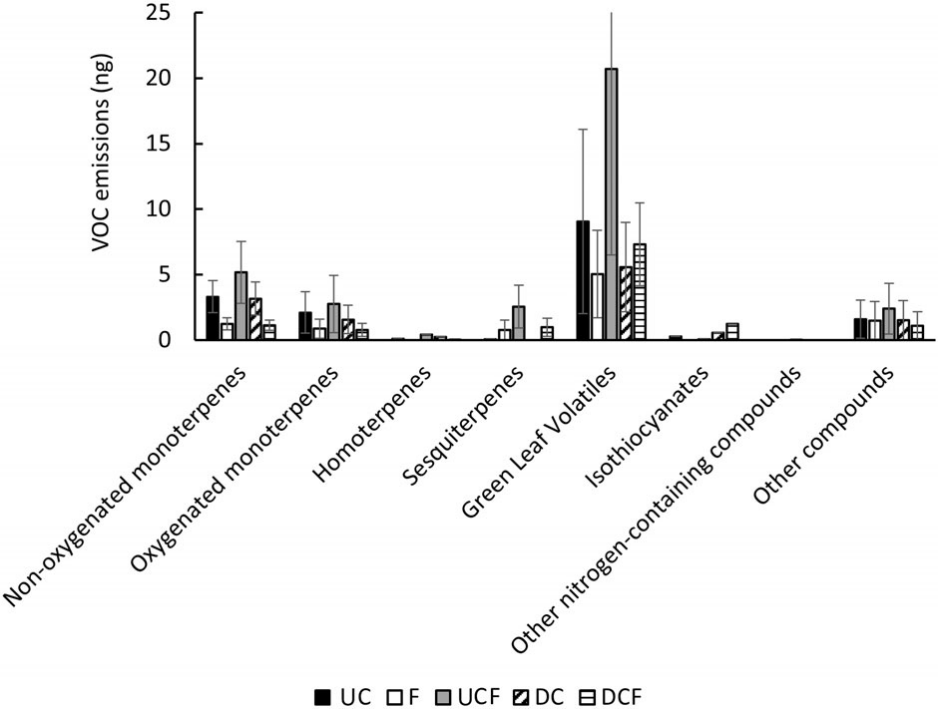
Average VOC emissions by group of molecules from all treatments (C–F, UCF–UC, UCF–DC, and DCF–C). Error bar in green leaf volatiles treatment UCF was cut to scale the figure.

A total of 32 VOCs were detected from the five treatments. These were grouped as green leaf volatiles (GLV), homoterpenes, non‐oxygenated monoterpenes, oxygenated monoterpenes, and sesquiterpenes. The anova performed on the total volatile emissions across treatments did not indicate a significant difference between treatments (df = 4, *F* = 2.521, *P* = 0.059; Fig. 3). When each individual compound was analysed across the treatments, multiple significant differences were observed (Table 2).

**Fig. 4:**
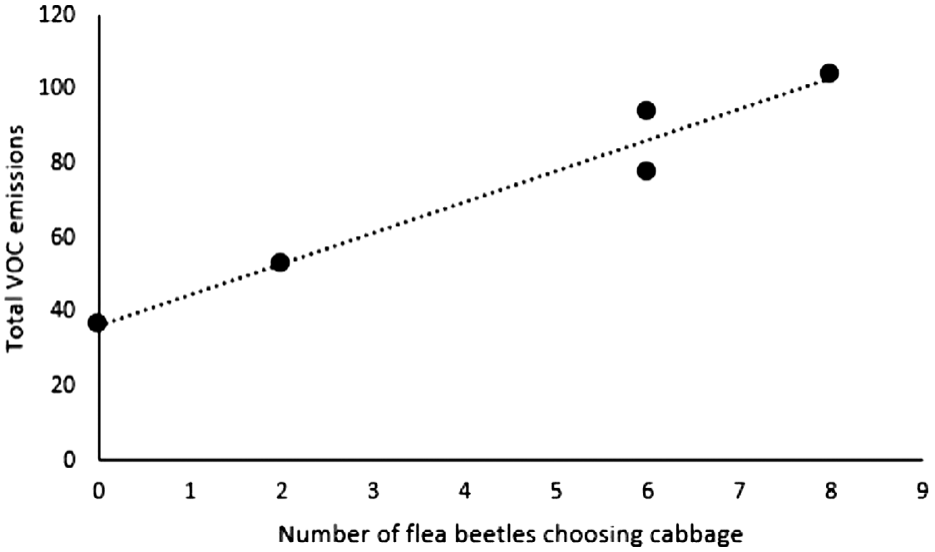
Correlation of total VOC emissions from undamaged cabbage (UC) and number of times flea beetles chose this experimental plant over faba bean (F).

The emitted VOCs from each treatment were analysed in pairs, comparing the specific VOCs to which flea beetles were individually exposed in the Y‐tube. Between UC and F, there were no significant differences in any of the detected VOCs. However, in the case of flea beetles choosing between UC and F, there was a correlation between higher VOC emissions in cabbage and a larger number of flea beetles choosing undamaged cabbage (Table 3, Fig. 4). Between UCF and UC, UC emitted significantly more β‐pinene (N = 10, U = 25.000, SE = 4.787, *P* = 0.008) and (*Z*)‐sabinene‐hydrate (N = 10, U = 24.000, SE = 4.787, *P* = 0.016) than UCF. However, UCF emitted significantly more (*E*)‐caryophyllene (N = 10, U =< 0.001, SE = 4.488, *P* = 0.008) than UC. Between DC and UCF, UCF emitted significantly more (*E*)‐β‐ocimene (N = 10, U = 22.500, SE = 4.249, *P* = 0.032), methyl salicylate (N = 10, U = 25.000, SE = 4.640, *P* = 0.008), (*E*)‐caryophyllene (N = 10, U = 25.000, SE = 4.488, *P* = 0.008), and α‐humulene (N = 10, U = 22.500, SE = 4.249, *P* = 0.032) than DC. Between DCF and UC, UC emitted more α‐thujene (N = 10, U =< 0.001, SE = 4.787, *P* = 0.008), α‐pinene (N = 10, U = <0.001, SE = 4.787, *P* = 0.008), sabinene (N = 10, U =< 0.001, SE = 4.787, *P* = 0.008), β‐myrcene (N = 10, U =< 0.001, SE = 4.787, *P* = 0.008), α‐terpinene (N = 10, U = 2.000, SE = 4.729, *P* = 0.032), limonene (N = 10, U = 2.000, SE = 4.787, SE = 4.787, *P* = 0.032), 1‐8‐cineole (N = 10, U =< 0.001, SE = 4.787, *P* = 0.008), γ‐terpinene (N = 10, U = 1.000, SE = 4.787, *P* = 0.016), and (*Z*)‐sabinene hydrate (N = 10, U =< 0.001, SE = 4.773, *P* = 0.008) than DCF. DCF emitted more 1‐octen‐3‐ol (N = 10, U = 25.000, SE = 4.488, *P* = 0.008) and (*E*)‐caryophyllene (N = 10, U = 25.000, SE = 4.640, *P* = 0.008) than UC.

**Table 3:** Total VOC emissions from cabbages in each plant combination of the undamaged cabbage–faba bean olfactory assay. Each replicate consisted of 10 flea beetle individuals.

replicate number	number of flea beetles choosing cabbage	total VOC emission of cabbage (ng)
1	2	53.03775
2	6	77.36196
3	0	36.38259
4	8	104.0467
5	6	93.75594

## DISCUSSION

In this laboratory study, flea beetles orientated significantly more frequently towards the unmasked volatiles of cabbage, mimicking monocropping, over the cabbage mixed with faba bean, mimicking strip cropping. Surprisingly, despite being a cabbage pest with no recorded activity on faba bean, when presented with a single cabbage plant versus a single faba bean plant, flea beetles orientated towards both equally. This unexpected observation could be due to the low VOC emissions of undamaged cabbage in replicates 1 and 3 of the undamaged cabbage–faba bean comparison. Further corroboration of this explanation was provided by a correlation between the quantity of total VOC emissions from undamaged cabbage and the number of flea beetles orientating towards the undamaged cabbage odour (Fig. 4). Importantly, our experimental design allowed us to examine the precise emissions of each odour source combination used in the olfactory assays. For two out of the five odour source pairs used in this test (replicates 1 and 3), flea beetles chose faba bean over undamaged cabbage. It is notable that in these two replicates, the VOC emissions of the cabbages were low (Table 3), potentially not providing a suitably attractive cue to the flea beetles, and possibly skewing the data. This also highlights the importance of sampling VOCs from the exact plants to which the insects are exposed during an olfactory experiment. Conventionally, orientation is compared to separately sampled VOCs from the plants, which are the same species and cultivar; however, individuality of the plants can be lost using this procedure.

In the DBM behavioural assays, the effect of combining the two crop plants on orientation was not as clear. Given a choice between cabbage and faba bean, DBM chose cabbage with a significantly greater frequency, as predicted. However, when cabbage was combined with faba bean and the alternative was cabbage alone, DBM did not show any preference between the treatments. Plant volatiles emitted by faba bean have been shown to attract DBM (Gillott *et al*. 1986), and the adult DBM can utilize extrafloral nectar produced by faba bean as a nutrient source (Winkler *et al*. 2005), which could explain the observed lack of a masking effect of faba bean. To further investigate which individual compounds in the blend could be responsible for the diamondback moth's orientation, we performed a headspace analysis on cabbage, faba bean, and their mixture with and without foliar damage to the cabbage. However, the headspace analysis in the DBM orientation experiment was not performed on the same individual plants used in the behavioural analysis and hence does not allow matching precise behavioural observations to the VOC blends collected. Nevertheless, a comprehensive analysis of the typically emitted VOCs was achieved. We detected significantly higher concentrations of (*Z*)‐3‐hexenyl acetate and (Z)‐3‐hexen‐1‐ol in the faba bean–damaged cabbage mix DCF, compared to the UC and DC treatments. These compounds are both known attractants of DBM (Yan *et al*. 2023), which could explain why the combination of cabbage and faba bean was as attractive to DBM as the single cabbage. This could mean that relying only on masking VOC cues in intercropping cabbage and faba bean will not protect against DBM. Flea beetles are not known to utilize extrafloral nectar in their diet, which consists mostly of leaves and stems as adults and roots as larvae and could be why we observed a strong masking effect of faba bean in VOC mixes. It is common that one crop combination cannot reduce all pest insect incidence equally, and in some cases, pest incidence will even be increased with intercropping (Huss *et al*. 2022).

Damaged cabbage attracted DBM more than undamaged cabbage, which shows that protecting the cabbage from pre‐existing damage is vital in controlling this pest, whereas foliar damage did not affect the orientation of flea beetles. In the flea beetle orientation experiment, the pests had only VOC cues and no visual cues available to orientate towards their target plant. Further, the plant VOC emissions were recorded after each replicate to account for the variation in VOC emissions even between the same treatment type, but also to correlate the VOC emissions to pest orientation. In the pairwise analysis, we identified 18 occasions with 15 compounds, where VOC emissions differed significantly between the two treatments to which flea beetles were exposed while undergoing the olfactory experiment. Consistent with the findings of Gruber *et al*. (2009), our pairwise olfactory orientation experiments showed that flea beetles were less attracted to plants emitting higher levels of (*E*)‐caryophyllene, β‐pinene, and limonene compared to the adjacent plant in the bioassay setup. In contrast, plants that emitted higher levels of sabinene attracted more flea beetles compared to the adjacent plant in the bioassay. Notably, there were instances where we observed significant differences in emissions of these compounds that coincided with distinct orientations of flea beetles, favouring treatments with lower levels of unattractive compounds and higher levels of attractants. Also (*E*)‐β‐ocimene was a strong attractant of *Phyllotreta cruciferae*, a species of flea beetle. However, in our results comparing UCF to DC, we found significantly higher emission of (*E*)‐β‐ocimene in UCF, but significantly higher orientation towards DC. The orientation of *P. cruciferae* was assesed using one VOC at a time, whereas in our setup the pests were exposed to the full blend of VOCs released by the experimental plants. Investigating the full blend of VOCs emitted by the plants is important to understand how the insects orientate under field conditions. Our results support the findings by Gruber *et al*. (2009), even when flea beetles were exposed to the full blend. Identifying the specific plant VOCs responsible for attracting pest insects to crops can prove a powerful tool in establishing pest control methods. For these methods to be applied in field conditions, plant chemical diversity and the diversity among plant species must be considered, as both have direct effects on insects that rely on olfactory orientation while foraging.

## CONCLUSION

This laboratory study investigated the potential impact of mixing VOCs emitted by cabbage and faba bean on the orientation of two major pests of cabbage, namely flea beetles and DBM. While the presence of faba bean had a clear effect in redirecting flea beetles towards unmasked volatiles of cabbage, the effect on DBM was less evident. The lack of a masking effect of faba bean could be related to the higher concentration of (*Z*)‐3‐hexenyl acetate and (*Z*)‐3‐hexen‐1‐ol in the cabbage–faba bean mixtures. Second, the attractive quality of extrafloral nectar for DBM could explain this behaviour and merits further study. The aversion of flea beetles for the faba bean–cabbage combination over cabbage alone showed that when pests are guided only by VOC emissions, this control strategy is effective in masking the targeted crop. Our results do not reflect field conditions, and further study is needed to validate the potential positive effect of stripcropping cabbage and faba bean to control flea beetles. Nonetheless, these findings contribute valuable insights into a broader understanding of plant–insect interactions in complex odour environments, especially since abundant use of insecticides can lead to rising levels of resistance in pest insect populations.

## Author Contributions

J.K.M. was the lead author of the manuscript, planned and conducted the olfactory orientation experiments, collected and analyzed the VOC samples, and performed the data analysis. S.S. assisted with the planning of the olfactory experiments and co‐wrote the manuscript. H.R. contributed to the experimental planning and co‐wrote the manuscript. E.R. assisted with the olfactory orientation experiments. J.D.B. contributed to the planning of the olfactory experiments and co‐wrote the manuscript.
